# Searching joint association signals in CATIE schizophrenia genome-wide association studies through a refined integrative network approach

**DOI:** 10.1186/1471-2164-13-S6-S15

**Published:** 2012-10-26

**Authors:** Peilin Jia, Zhongming Zhao

**Affiliations:** 1 Department of Biomedical Informatics, Vanderbilt University School of Medicine, Nashville, TN, USA; 2 Department of Psychiatry, Vanderbilt University School of Medicine, Nashville, TN, USA; 3 Department of Cancer Biology, Vanderbilt University School of Medicine, Nashville, TN, USA; 4 Center for Quantitative Sciences, Vanderbilt University School of Medicine, Nashville, TN, USA

## Abstract

**Background:**

Genome-wide association studies (GWAS) have generated a wealth of valuable genotyping data for complex diseases/traits. A large proportion of these data are embedded with many weakly associated markers that have been missed in traditional single marker analyses, but they may provide valuable insights in dissecting the genetic components of diseases. Gene set analysis (GSA) augmented by protein-protein interaction network data provides a promising way to examine GWAS data by analyzing the combined effects of multiple genes/markers, each of which may have only individually weak to moderate association effects. A critical issue in GSA of GWAS data is the definition of gene-wise *P *values based on multiple SNPs mapped to a gene.

**Results:**

In this study, we proposed an alternative restricted search approach based on our previously developed dense module search algorithm, and we demonstrated it in the CATIE GWAS dataset for schizophrenia. Specifically, we explored three ways of computing gene-wise *P *values and examined their effects on the resultant module genes. These methods calculate gene-wise *P *values based on all the SNPs, the top ranked SNPs, or the most significant SNP among all the SNPs mapped to a gene. We applied the restricted search approach and identified a module gene set for each of the gene-wise *P *value data set. In our evaluation using an independent method, ALIGATOR, we showed that although each of these input datasets generated a unique set of module genes, all of them were significant in the GWAS dataset. Further functional enrichment analysis of these module genes showed that at the pathway level, they were all consistently related to neuro- and immune-related pathways. Finally, we compared our method with a previously reported method.

**Conclusion:**

Our results showed that the approaches to computing gene-wise *P *values in GWAS data are critical in GSA. This work is useful for evaluating key factors in GSA of GWAS data.

## Background

Genome-wide association studies (GWAS) have emerged as a powerful tool to examine the genetic components of complex disease. During the past six years, GWA studies have successfully uncovered a few thousands of markers/genes that are associated with complex diseases/traits [1]. So far, most standard GWA studies have focused on single marker based analysis and applied the genome-wide significance cutoff *P *value 5 × 10^-8 ^for detecting significant markers; however, many weakly or moderately associated markers (e.g., whose *P *values are between 0.05 and 5 × 10^-8^) may also provide valuable insights. These markers have been generally missed in the standard analysis.

Gene set analysis (GSA) of GWAS data provides an alternative approach of assessing the joint effects of multiple genes [2], regardless of whether they are individually significant or not. Complex diseases are likely caused by multiple genes and markers, each of which may only contribute weak to moderate effect. Given that these markers are biologically or functionally correlated, GSA would increase the power to detect them in a typical GWAS dataset [2]. GSA typically uses pathways or functional categories of cellular processes to define gene sets, such as those from the KEGG database [3] and the Gene Ontology (GO) annotations [4]. Among the available GSA methods, representative ones include the Gene Set Enrichment Analysis (GSEA) of GWAS data [5], the Association LIst Go AnnoTatOR (ALIGATOR) [6], and the Gene set Ridge regression in ASsociation Studies (GRASS) [7]. An advanced GSA approach is to use protein-protein interaction (PPI) network data as the platform to dynamically search for "gene sets", namely network modules, and perform an enrichment test for association signals. Our dense module search of GWAS association signals from PPI network is one of the first methods[8]. Besides, Rossin et al. [9] used PPI network to assess whether the association loci uncovered in standard GWA studies are significantly connected through PPIs. They adapted a straightforward way of defining subnetworks [9], i.e., given a list of loci and the genes located in them, "direct networks" and "indirect networks" are constructed based on the network data. However, the GWAS data is not effectively incorporated in the process of network building such that the moderately associated genes (0.05 ~ 5 × 10^-8^) are still missed. Therefore, more comprehensive methods are in need to incorporate the GWAS data with the PPI data to help construct, prioritize, and evaluate subnetworks for complex diseases.

In most of these GSA methods, a critical issue is how to define the gene-wise *P *value, the *P *value representing the association signal at each gene region. In a typical GWAS data, statistics at the single nucleotide polymorphism (SNP) level is used, but biological data (pathways or PPI network) are typically annotated to genes/proteins. Thus, there is a gap between marker's significance and gene-wise significance. A popularly applied method in the field is to select the most significant SNP from the multiple SNPs mapped to each gene and represent the gene by the smallest *P *value [2,5,10]. Although this method is sensitive to retain the association signals and is easy to implement, this way of using the minimum *P *value bears intrinsic biases, including gene length, SNP density, and/or linkage disequilibrium (LD) structures[11]. Recently, several reported methods aim to compute gene-wise *P *values, such as GATES [12], which adapts the Simes' test within each gene, VEGAS [13], which builds on the multivariate normal distribution and takes into account pairwise LD values, and the SNP-set analysis [14]. Incorporation of these methods into a gene set analysis of GWAS data can reduce potential biases at the gene level and improve the robustness of follow up analyses.

In this work, we proposed a restricted search strategy to implement our previously developed dense module search (DMS) method [8]. This new strategy could greatly reduce the computational intensity problem. We demonstrated this method in a GWAS dataset for schizophrenia and explored three different ways to define gene-wise *P *values. Our results showed that the way to define gene-wise *P *values could affect the network-based analysis substantially, and it also concluded that caution is needed when designing and interpreting the results.

## Materials and methods

### GWAS dataset

We used the GWAS dataset for schizophrenia from the Clinical Antipsychotic Trial of Intervention Effectiveness (CATIE) project [15]. The CATIE project is a multiphase randomized controlled trial initially designed to investigate and improve the use of antipsychotic medications in treating schizophrenia patients. We included the samples involving 738 schizophrenia patients and 733 controls, which were genotyped by Perlegen Sciences using the Affymetrix 500K and Perlegen's custom 164K chip, resulting in ~446 k genotyped SNPs. A detailed description of the samples can be found in reference [15]. We accessed this dataset (Distribution 7.0) from http://www.nimhgenetics.org/ through NIMH approval. Only the Caucasian samples were used. We followed the pipeline of quality control, including the selection of samples and markers, as described in references [8,16,17].

### Gene-wise *P *value

To compute gene-wise *P *values, given multiple SNPs mapped to each gene, an ideal algorithm should account for potential confounding factors, such as gene length, SNP density, and LD structures. We incorporated the software tool VEGAS [13] to compute the gene-wise *P *values. VEGAS combines the information of multiple SNPs by making use of simulations from the multivariate normal distribution while explicitly accounting for the LD between markers. We followed the default settings in VEGAS; this default setting maps SNPs to genes with 50 kb extension of gene boundaries. The HapMap CEU samples (http://www.hapmap.org/, release R2) were selected to estimate the LD structure in our work, as we only included the Caucasian samples in the CATIE data.

We explored three options in VEGAS to compute gene-wise *P *values based on sets of SNPs: (1) using all the SNPs mapped to a gene (hereafter denoted as "VEGAS-all"); (2) using the top 10% SNPs based on SNP-level *P *values ("VEGAS-top"); and (3) using the most significant SNP, i.e., the SNP with the smallest *P *value ("minP"). Note that the minP option is the same as the smallest *P *value strategy widely used in many post-GWAS analysis methods. We included it here to compare with the other, more advanced approaches based on combining of multiple SNPs.

### PPI dataset

Our background PPI network data were collected from six public PPI databases: MINT, IntAct, DIP, BioGRID, HPRD, and MIPS/MPact. We downloaded these data from the Protein Interaction Network Analysis (PINA) platform (March 2010) [18]. To ensure the reliability of the PPI data, we explicitly included only interactions that have experimental evidence and involve both interactors from human genes. Self-interaction and duplicates were removed. The final network included a total of 10,377 nodes and 50,109 interactions.

### Module search algorithm

We first overlaid the GWAS data onto the whole human PPI network by assigning each node a *z*-score: $z=\Phi^{-1}\left(1-P\right)$, where $\Phi^{-1}$ is the inverse normal cumulative density function and *P *is the gene-wise *P *value from any one of the three methods. For a module with *k *genes, a module score was computed according to the Stouffer's *Z*-score method:$Z_{m}=\sum_{i=1}^{k}z_{i}/\sqrt{k}$. The detailed module construction process can be found in our previous work [8]. Briefly, for a given "seed node", a "best module" with the maximum module score will be returned in the context of the working parameters, i.e., *d*=2 and *r*>0.1. Here, *d *is distance for the nodes to be included to the seed node and *r *is the network score increment cutoff value.

The overall network weighted by the GWAS data serves as the working background. The restricted search strategy we proposed in this study is implemented as follows. First, we ranked all the nodes in the network according to their weights *z_i _*and started with the node that had the highest score as the seed to perform a module search. Once the module was generated, all the nodes present in this module, including the seed node itself, were then removed from the network, and the rest of the network constituted the new background network. A new module search round was started again, with the highest scored node in the new background network each time, until none of the nodes in the background network could generate a module with ≥5 nodes (i.e., the minimum number of nodes we required to define a module). This restricted strategy takes into account every single node in the background network but does not require using each of them as a seed node, which has been implemented in our previous work. Thus, this tactic could greatly reduce the computational duty and also avoid the heavy redundancy of resultant modules using our original algorithm.

### Significance test

To estimate the significance of the resultant modules, we calculated *P *values based on the module scores (*Z_m_*). We adopted the method proposed in [19] to empirically estimate the null distribution, which is assumed to be a normal distribution. Specifically, we used the median-centered module score to estimate the location parameters δ and σ for the empirical null distribution using the R package *locfdr *and computed standardized module scores by *Z_S _*= (*Z_m_'*- δ)/σ, where *Z_m_' *is the median-centered module score. The final module *P *values were obtained using the standard calculation, *P*(*Z_m_*) = 1-Φ(*Z_S_*), where Φ is the normal cumulative density function. The module *P *values were then used for significance test of the resultant modules and help module selection.

### Evaluation by ALIGATOR

We utilized the software tool ALIGATOR [6] to evaluate the module gene sets. The algorithm of ALIGATOR is initially designed to prioritize Gene Ontology (GO) categories using summary GWAS data at the SNP level. Building on a resampling strategy, ALIGATOR first pools all the SNPs and their *P *values from the GWAS data and builds the SNP collection. In each resample, the algorithm randomly selects SNPs from the collection and records the number of significant genes defined by the selected SNPs. Here, significant genes were defined as those with at least one SNP that has a *P *value less than a pre-defined cutoff value, e.g., 0.05. The random selection process keeps running until the significant genes targeted by the selected SNPs reaches the number of the significant genes in the real case. After the resampling SNP set is constructed, each of the GO categories are compared with the resample data to obtain the number of significant genes. This resampling process is repeated numerous times (e.g., 50,000), resulting in the null distribution of the number of significant genes in each GO category. Finally, an empirical *P *value can be computed for each GO category by comparing the significant genes in the category in the real case versus those in the resample sets.

In our application, instead of using GO categories, we constructed new gene sets based on the module genes. The module genes identified by each input dataset were pooled as one gene set, and 3 module gene sets were generated, corresponding to the input dataset of VEGAS-all, VEGAS-top, and minP. As a comparison, we also included the KEGG pathways (downloaded as of March, 2011) [3]. We restricted the KEGG pathway size (number of genes in a pathway) to ≥5 and ≤300. In total, there were 204 KEGG pathways plus 3 module gene sets collected for the ALIGATOR analysis. We followed the ALIGATOR default definition of significant genes, i.e., genes with at least one SNP having *P* <0.05, and we performed the resampling 10,000 times. Multiple testing correction by the Benjamini & Hochberg (BH) method [20] was then conducted.

### Comparison with DAPPLE

We compared with another available network based GSA method, "Disease Association Protein-Protein Link Evaluator (DAPPLE)" [9]. We applied DAPPLE to the CATIE dataset. DAPPLE aims to evaluate whether genes in association loci are significantly connected by PPI, where the association loci are typically defined by the standard single marker analysis of a GWAS dataset for a complex disease/trait. Using the genes located in these association loci, DAPPLE searches for two types of subnetworks: a direct network, in which the input genes are directly connected, and an indirect network, in which the input genes are connected through a common interactor [9]. Both networks are then evaluated by a permutation test to assess their significance. Because the construction process of the resultant subnetworks starts with the input association genes/loci, the method relies heavily on the input genes and can generate different subnetworks if the input gene/locus list is changed. However, the ways to define associated genes have not been standardized yet, although all adopt a pre-defined hard cutoff. For example, the widely applied method employs the cutoff of 5 × 10^-8^, which is challenging in psychiatric diseases, because the association signals are typically weak in psychiatric GWAS datasets. Alternatively, a user-defined cutoff value, which can be less strict yet arbitrary, could be considered.

### Functional evaluation

We performed pathway enrichment test of the three module gene sets using the canonical KEGG pathways. The hypergeometric test was implemented with the genes in the network used as the gene universe and module genes used as the genes of interest. Multiple testing correction was performed using the Bonferroni method.

## Results

### Exploration of gene-wise *P *values

**Figure 1:**
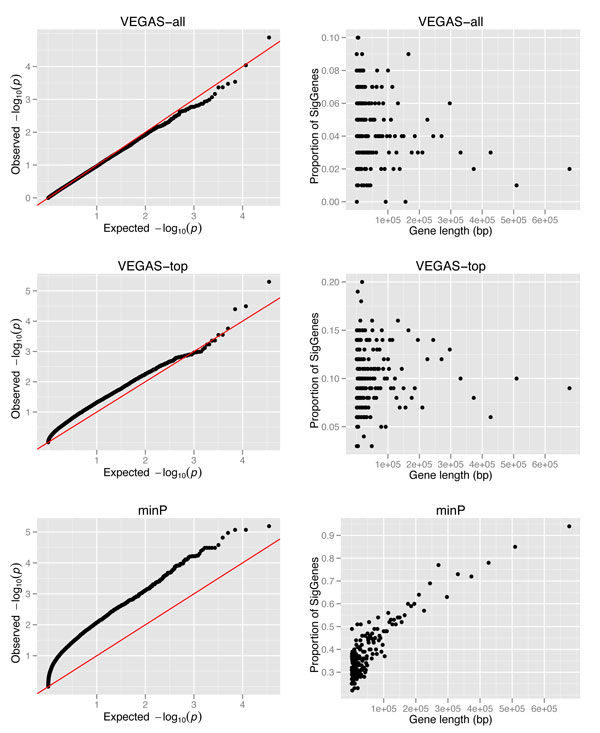
**Q-Q plot of gene-wise *P *values and their distribution versus gene length**. The three rows of panels represent the method of VEGAS-all, VEGAS-top, and minP, respectively. In each row (method), the left panel displays the Q-Q plot, while the right panel shows the distribution of gene-wise *P *values versus gene length. In the right panel, gene length (X-axis) was separated to bins, each of which has 100 genes; the Y-axis shows the proportion of significant genes (denoted as "SigGenes") in each length bin. SigGenes were defined as those with gene-wise *P *values <0.05.

Using VEGAS, three sets of gene-wise *P *values were generated, each of which used the full set of SNPs, the top 10% of SNPs, and the most significant SNP among all the SNPs mapped to a gene. Figure 1 shows the Q-Q plot of each of these *P *value sets as well as the distribution of gene-wise *P *values versus gene length. The Q-Q plot of the VEGAS-all set showed a trend most closely located as would be expected in random cases. Furthermore, this set did not display any strong correlation with gene length (Figure 1), indicating that the VEGAS-all set does not have strong confounding factors. On the other hand, the minP method generates the most inflated distribution, and the gene-wise *P *values showed a strong correlation with gene length, i.e., higher proportion of significant genes were observed with larger gene length (Figure 1). The behavior of the VEGAS-top set falls between those of the other two methods: neither the Q-Q plot nor the correlation with gene length showed strong inflation.

### Dense module search for schizophrenia

For each of the three gene-wise *P *value sets, we applied the restricted search strategy of DMS to construct candidate modules significantly enriched for schizophrenia. The restricted strategy greatly reduced the space for available seed nodes; thus, the executing time for a complete search round is much shorter than the full search strategy. In each set, we obtained the number of modules, the number of significant modules, and the number of module genes in the significant modules (Table 1).

**Table 1:** Summary of the module search results by the strict searching strategy

Gene-wise method	# modules	# significant modules	# module genes
VEGAS-all	256	13	144
VEGAS-top	572	33	323
minP	703	36	319

To explore the results, we first compared the module genes in each data set using the weighted DMS method. As shown in Figure 1, the overlapped genes varied greatly among the three data sets, indicating that the gene-wise *P *value definition approach influenced the resultant subnetworks substantially. When using the VEGAS-all method, the smallest number of module genes were generated, as well as the number of significant modules, compared to the other methods, while the minP method generated the largest number of significant modules and module genes. However, the minP method is prone to potential biases, such as gene length, SNP density and LD structure. Thus, the results in this minP set might be inflated by these biases.

### Validation by ALIGATOR

For each of the three resultant sets, we validated them using ALIGATOR, along with the canonical KEGG pathways. As shown in Table 2, the three resultant module gene sets, each of which contains module genes identified using the VEGAS-all, VEGAS-top, and minP gene-wise *P *values as node weights, showed the most significant *P *values, especially when compared to KEGG pathways. This indicates that all three gene sets were significantly enriched with association signals for schizophrenia, regardless of the methods to define gene-wise *P *values. Interestingly, the significant pathways from KEGG include several immune-related pathways, such as asthma (hsa05310, *P*=8.0 × 10^-4^), cytokine-cytokine receptor interaction (hsa04060, *P*=2.9 × 10^-3^), and intestinal immune network for IgA production (hsa04672, *P*=5.5 × 10^-3^). These findings confirm the previous knowledge that schizophrenia is a complex disease involving immune systems [11,21-24].

**Table 2:** Analysis results of module gene sets and KEGG pathways by ALIGATOR (*P_BH _*<0.2)

Gene set	*P*	***P_BH_***^$^
VEGAS-all module genes	1.0 × 10^-4^	0.007
VEGAS-top module genes	1.0 × 10^-4^	0.007
minP module genes	1.0 × 10^-4^	0.007
Asthma (hsa05310)	8.0 × 10^-4^	0.040
Tryptophan metabolism (hsa00380)	1.7 × 10^-3^	0.068
Gap junction (hsa04540)	2.3 × 10^-3^	0.076
Cytokine-cytokine receptor interaction (hsa04060)	2.9 × 10^-3^	0.082
Intestinal immune network for IgA production (hsa04672)	5.5 × 10^-3^	0.137

^$^*P *values were adjusted by Benjamini & Hochberg (BH) method [20].

### Exploration of module genes

**Figure 2:**
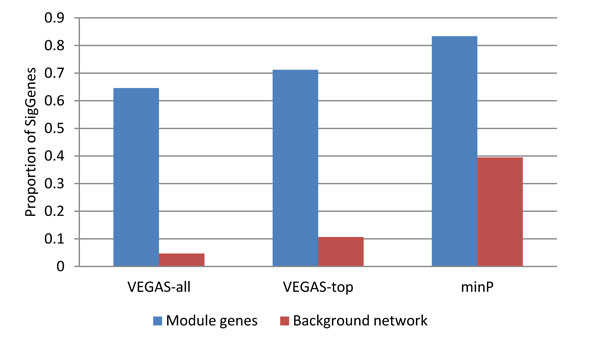
**Proportion of significant genes in each module of gene sets**. Significant genes were defined as those with gene-wise *P *values <0.05. The proportion of significant genes in module genes and in the background genes in the network were shown for each scenario of using VEGAS-all, VEGAS-top, and minP as input genes, respectively.

At the gene level, module genes showed extreme convergence of significant genes compared to the background genes in the network. Defining significant genes as those whose gene-wise *P *values were less than 0.05, we computed the proportion of significant genes in the module gene set and the background set for each of the three input datasets. As shown in Figure 2, the module gene sets had a high proportion of significant genes in all three scenarios, with the VEGAS-all dataset showing the largest difference. Note that the minP dataset of gene-wise *P *values is possibly inflated (Figure 1), and consistently, the minP weighted background network has a higher proportion of significant genes compared to the other two scenarios. In such a context, the high proportion observed in minP module genes (83.4%, Figure 2) should be interpreted with caution.

We observed several well-studied candidate genes for schizophrenia in each of the three resultant module gene lists, such as *AKT1*, *DTNBP1*, *ESR1*, *FLNB*, *GRIN1*, *IL4*, *NOS1AP*, *YWHAZ*, and others. When compared to the genes previously studied for associations with schizophrenia as those deposited in the SzGene database [25], a total of 27 module genes were found to have positive association results (Table 3). Further examination of these genes showed that only a few of them were simultaneously identified by all three gene-wise *P *value definition methods, while many genes were uniquely identified in only one of the three scenarios (the second column in Table 3). The diversity of the module genes suggests that each of these approaches to define gene-wise *P *values may have its own advantages in prioritizing candidate genes for the disease, yet the methods also differ greatly in the resultant gene sets.

**Table 3:** Summary of module genes having positive association results in previous studies

Gene	Module gene set^$^	# SNPs	**Chr**.	Start (bp)	Stop (bp)	*P*_VEGAS-all_	*P*_VEGAS-top_	*P*_minP_
*NOS1AP*	a, m	125	1	160306204	160606437	0.005	0.007	1.07 × 10^-4^
*PTGS2*	t, m	14	1	184907591	184916179	0.013	8.5 × 10^-4^	1.07 × 10^-5^
*TSNAX*	t	11	1	229731021	229768892	0.476	0.306	0.113
*DISC1*	m	106	1	229829183	230243641	0.349	0.100	0.004
*FLNB*	a	47	3	57969166	58133017	0.030	0.034	0.007
*GSK3B*	t	40	3	121028235	121295203	0.141	0.073	0.011
*PDE4D*	m	193	5	58300622	59225378	0.277	0.018	6.16 × 10^-5^
*IL4*	a	12	5	132037271	132046267	0.069	0.019	0.007
*DTNBP1*	a	52	6	15631017	15771250	0.020	0.022	0.008
*RPP21*	t, m	48	6	30420915	30422611	0.014	0.001	1.04 × 10^-4^
*LTA*	m	43	6	31648071	31650077	0.450	0.075	0.003
*TNF*	m	43	6	31651328	31654091	0.598	0.184	0.003
*ESR1*	a, t, m	86	6	152053323	152466101	0.091	0.027	0.004
*TBP*	t	13	6	170705395	170723872	0.031	0.019	0.010
*EGFR*	t, m	72	7	55054218	55242525	0.556	0.294	0.026
*YWHAZ*	a, t, m	17	8	102000089	102034745	0.857	0.685	0.268
*GRIN1*	a, t, m	3	9	139153429	139183029	0.016	0.005	0.004
*ATE1*	m	59	10	123492614	123677536	0.491	0.155	9.28 × 10^-4^
*IFNG*	t, m	26	12	66834816	66839788	0.068	0.007	0.002
*NOS1*	m	45	12	116135361	116283965	0.643	0.315	0.028
*DGKH*	t	68	13	41520888	41701888	0.106	0.031	0.002
*AKT1*	a, t	6	14	104306731	104333125	0.076	0.056	0.026
*TP53*	t, m	11	17	7512444	7531588	0.540	0.219	0.075
*PRKCA*	t	129	17	61729387	62237324	0.995	0.857	0.029
*MAG*	m	13	19	40474877	40496547	0.097	0.037	0.003
*DNMT3B*	t, m	21	20	30813851	30860823	0.026	0.002	1.75 × 10^-4^
*MYH9*	m	37	22	35007271	35113927	0.050	0.017	5.75 × 10^-4^

**^$^**a: VEGAS-all; t: VEGAS-top; m: minP.

### Comparison with the existing method (DAPPLE)

DAPPLE requires a user-defined list of loci from the GWAS dataset. In our case, the CATIE GWAS dataset did not report any marker with significant *P *values at 5 × 10^-8 ^[15], and no genes could be used for the DAPPLE method at this conventional definition. Therefore, we manually selected the top 30 genes in each of the three gene-wise data sets, regardless of cutoff values for significance. Table 4 summarized the results using DAPPLE analysis. In the scenario of VEGAS-all top genes, a total of 4 direct interactions involving 5 genes were identified. Six genes were prioritized that achieved a corrected *P *value <0.05, all of which belong to the group of 30 input genes. In contrast, 18 of the 30 input genes were included in our module gene list. In the scenario of VEGAS-top genes, only one direct interaction and two prioritized genes were reported. Meanwhile, 21 of the 30 genes were found in the module gene list. Finally, in the scenario of minP genes, three direct interactions and three prioritized genes were reported, while 19 of the 30 genes were found in our module gene list. These results indicated that our DMS methods could effectively recruit significant genes as compared to DAPPLE.

**Table 4:** Comparative results by DAPPLE for the top 30 genes in VEGAS-all, VEGAS-top, and minP data sets

	VEGAS-all	VEGAS-top	minP
*DAPPLE*			
# of direct interactions	4	1	3
# genes to prioritize	6	2	3
Mean associated protein direct connectivity	1.6	1.0	2.0
Mean associated protein indirect connectivity	58.1	6.1	7.5
*DMS*			
# module genes	18	21	19

### Functional enrichment test of the module genes

Our functional enrichment test of the module gene sets showed that the neurotrophin signaling pathway (hsa04722) was significantly enriched in all three gene sets (Table 5). The neurotrophin pathway functions in the differentiation and survival of neural cells, playing significant roles in neural development. A total of 10, 17, and 18 module genes from the three module gene sets, VEGAS-all, VEGAS-top and minP, respectively, were presented in this pathway. However, the diversity at the gene level was high: only 3 genes were overlapped among all three module gene sets in this pathway, further supporting the rationality of functional analysis at the gene set level. Interestingly, in the minP module gene set, there were also two immune-related pathways significantly enriched: the T cell receptor signaling pathway (hsa04660) and the antigen processing and presentation pathway (hsa04612).

**Table 5:** Enriched KEGG pathways for module genes using the hypergeometric test

Pathway name (KEGG ID)	Pathway size	# module genes	*P*	*P*_Bonferroni_
*VEGAS-all*				
Neurotrophin signaling pathway (hsa04722)	116	10	6.96 × 10^-5^	0.007
Adipocytokine signaling pathway (hsa04920)	55	6	6.34 × 10^-4^	0.065
Cell cycle (hsa04110)	114	8	1.56 × 10^-3^	0.158
Chronic myeloid leukemia (hsa05220)	70	6	2.28 × 10^-3^	0.227
Vasopressin-regulated water reabsorption (hsa04962)	34	4	4.10 × 10^-3^	0.405
				
*VEGAS-top*				
Cell cycle (hsa04110)	114	20	8.61 × 10^-8^	1.15 × 10^-5^
Neurotrophin signaling pathway (hsa04722)	116	17	1.14 × 10^-5^	1.51 × 10^-3^
Endometrial cancer (hsa05213)	49	8	1.31 × 10^-3^	0.173
RIG-I-like receptor signaling pathway (hsa04622)	50	8	1.50 × 10^-3^	0.196
Chronic myeloid leukemia (hsa05220)	70	9	3.69 × 10^-3^	0.479
				
*minP*				
T cell receptor signaling pathway (hsa04660)	104	17	2.68 × 10^-6^	3.43 × 10^-4^
Neurotrophin signaling pathway (hsa04722)	116	18	2.91 × 10^-6^	3.69 × 10^-4^
Antigen processing and presentation (hsa04612)	59	12	8.58 × 10^-6^	1.08 × 10^-3^
Chronic myeloid leukemia (hsa05220)	70	13	1.03 × 10^-5^	1.28 × 10^-3^
Non-small cell lung cancer (hsa05223)	51	11	1.14 × 10^-5^	1.42 × 10^-3^

## Discussion

By taking advantage of our previously developed dense module search method, we proposed an alternative search strategy in this work and demonstrated it in the CATIE GWAS dataset, one of the major available GWAS datasets for schizophrenia. Additionally, we explored the different options to define gene-wise *P *values, including the VEGAS-all method, which built on all the SNPs mapped to a gene, the VEGAS-top method, which used the top 10% SNPs mapped to a gene, and the minP method, which used the most significant SNP. By applying our restricted search strategy in each of the three data sets, we showed that the VEGAS-all method generated the smallest number of module genes and was least affected by other potentially confounding effects such as gene length. The other two methods resulted in similar numbers of module genes. These results call for caution when selecting different methods to compute gene-wise *P *values, which may have significant influences on the resultant module genes prioritized for the disease.

The restricted search strategy is intended to reduce the overlap among modules. Assuming that a local environment of the background network includes 5 nodes, namely A, B, C, D, and E. Starting from node A, a module including A, B, C, and D would be generated at *Z_m+1_*>*Z_m_* × (1+*r*). Starting from B, a module including B, C, D, and E would be generated. In our previous strategy to apply DMS, both modules would be reported, even though they had 75% overlapping genes. In the current strategy, to resolve the issue of overlap, we starts with the node that has the highest weight, e.g., A, to search for the module. And then we would remove the module genes from the background network after it is done, e.g., the nodes A, B, C, and D would be removed from the network and, thus, from further analysis. In this way, the module starting from B would not be reported, as most nodes in it have already been removed from consideration. This ensures that each node in the network could be analyzed once and will be involved in only one module. Both methods have their own advantages. The traditional one performs a comprehensive search and allows every node in the network to have the chance of being a seed. The computational intensity is high and redundancy among modules is strong. Furthermore, the correlation among modules posts challenges for the follow up statistical tests when selecting modules. In contrast, the restricted strategy is computationally efficient by gradually shrinking the background network, and it ensures against physical overlap among modules. However, it may miss moderately significant genes that cannot be included in any module. In practice, either of the two strategies can be selected depending on the specific aims and project design.

Computation of gene-wise *P *values is one of the key steps in most post-GWAS analyses. There have been several methods and tools published to compute gene-wise *P *values. The most widely applied method in the field is to select the SNP with the smallest *P *value among all SNPs mapped to a gene, although this method is subjected to several known biases, such as gene length, SNP density, and the local LD structure. We selected VEGAS because of its advantages, such as acceptable computation time (<12 hours for a typical GWAS dataset like in our case) and no need of genotyping data. The rationale of including two formulations in VEGAS is that using all SNPs mapped to a gene (e.g., VEGAS-all method) is comprehensive but considering all SNPs potentially dilute the signals, while using part of the SNPs (e.g., VEGAS-top) may miss some informative SNPs but captures the most significant 10% SNPs for the computation.

However, VEGAS computes SNP-SNP matrix based on pairwise LD values and could only deal with autosomal SNPs. SNPs located on the sex chromosomes (X and Y) are not applicable for VEGAS and were removed from our network based analysis. Although these genes accounted for only a small proportion (3.9%) in the PINA network we used, more comprehensive algorithms that are able to handle all genes in the genome is needed for future work.

The module genes we identified, in any scenario, recruited neuro-related and/or immune-related genes and pathways. All three sets of module genes include well-studied candidate genes for schizophrenia (e.g., *DTNBP1*), glutamate receptors (e.g., *GRIN1*), several genes located in the MHC region (e.g., *HIST1H1A*, *HIST1H1C*, *HIST1H2AB*, *HIST1H2BB*, *HLA-E*), and genes from the 14-3-3 protein family (e.g., *YWHAQ*, *YWHAZ*). Interestingly, all three module gene sets contain several genes in the MHC region, even though none of these genes passed the significance test for single markers at 5 × 10^-8^. The MHC region has been shown to harbor significant association signals in a combinatory analysis of three GWAS datasets for schizophrenia [11,24]. The identification of these genes by our DMS method further confirmed this signal. It also proved that network based analysis could reveal markers that, although they individually failed the single marker test, their joint affects on the disease might be significant.

## Conclusions

We proposed an efficient network-assisted framework to identify candidate genes from GWAS data based on dense module search algorithm. Augmented by functional annotation as well as *a priori *knowledge about schizophrenia, we explored the methods to compute gene-wise *P *values based on multiple SNPs mapped to a gene and assessed their effects on downstream analysis. In specific applications, caution is needed when selecting different search strategies and methods for gene-wise *P *values. Future work to compute gene-wise statistics for all genes in the genome will further improve such applications.

## Competing interests

The authors declare that they have no competing interests.

## Authors' contributions

PJ and ZZ conceived and designed the experiments. PJ carried out the data analysis. PJ and ZZ drafted the manuscript. All authors read and approved the final manuscript.
